# Work-Family Conflict, Happiness and Organizational Citizenship Behavior Among Professional Women: A Moderated Mediation Model

**DOI:** 10.3389/fpsyg.2022.923288

**Published:** 2022-06-14

**Authors:** Ying Pan, Nadilai Aisihaer, Qinyi Li, Yue Jiao, Shengpei Ren

**Affiliations:** ^1^Economics and Management School, Nantong University, Nantong, China; ^2^International Business School Suzhou, Xi’an Jiaotong-Liverpool University, Suzhou, China; ^3^College of Business Administration, Shanghai Business School, Shanghai, China; ^4^ITO Bussiness Group, Sunyard Technology Co., Ltd, Hangzhou, China

**Keywords:** work-family conflict, family support, happiness, organizational citizen behavior, China

## Abstract

This study investigates the association between work-family conflict and organizational citizenship behavior and examines the mediated role of subjective happiness between and the moderated part of family support. A moderated mediation model is established based on the Conservation of Resources theory. We collected data from 386 employees of nine companies in China. This study shows that the work-family conflict of female professional employees is negatively correlated with organizational citizenship behavior, and that the relationship is mediated by subjective well-being. Furthermore, female professional employees’ family support moderates the effects of work-family conflict on subjective happiness and organizational citizenship behavior, with the relationship weaker when family support is higher. This study enriches the literature on work-family conflict by using family support as a mediating mechanism for work-family conflict. It enhanced our understanding of the influencing mechanisms of organizational citizenship behavior by constructing a more detailed model.

## Introduction

With the competition in the global market and the continuous growth of China’s economy (Cooke, 2013), women have gradually stepped into social work. Women gradually began to gain higher status and create more value in the public sphere (Chandra, 2012), which gradually blurred the traditional Chinese model of the division of labor between the two sexes: “males lead the outside, and females lead the inside.” Some women have achieved notable success at work (Koyuncu et al., 2012). However, with the continuous implementation of China’s multi-child policy. For most middle-class families, women need to earn income for the family and bear most of the housework and mental burden, which leads to career women getting caught in work-family conflict (Niles and Harris-Bowlsbey, 2017).

Previous studies have shown that female employees experience significant mood swings due to backlogs of work pressure (Sonja and Heidi, 1999), increasing family responsibilities, and an increasingly unbalanced work-life time (Beckett et al., 2015). Additionally, women tend to feel more stress in their family life, resulting in tension and other negative emotions (Lepine et al., 2002). However, these negative emotions are considered essential factors affecting the satisfaction of working women. As research into work and family issues increased, family support was identified as an important factor in reducing conflict (Kelley et al., 2021). However, family support refers to encouraging and helping each other based on the growth of each member’s physical and mental qualities to enhance the happiness of family members and ensure the regular operation of family relationships (Carr et al., 2008). Past research has found that family support is positively related to work-family affluence and negatively related to work-family conflict (Lee et al., 2013; Kelley et al., 2021). When stressful events occur at work and home (Staines, 1980), family support can effectively prevent female employees from being affected by negative emotions, properly handle stressful circumstances, and prevent work or family life from being disrupted by stressful events (Li et al., 2018). Family support ensures the emotional stability of female employees and further enhances their happiness (Cates et al., 2010). Literature reviews reveal that philosophers and psychologists have identified happiness (or, more broadly, subjective well-being) as one of the essential characteristics of a good life and a good society (Lin et al., 2021).

However, most theoretical perspectives ignore the importance of emotion in the work-family domain (Greenhaus and Powell, 2006). Too much past research has focused on employees’ work and family experiences, and little is known about the impact of work-family conflict on employee well-being (Lee D.-J. et al., 2018; Lee Y.-J. et al., 2018). Therefore, it is necessary to find a way to regulate employees’ living conditions, improve their well-being, and cope with the work-family crisis.

The current literature suggests that family responsibilities are a source of work-life conflict for women (Toffoletti and Starr, 2016). This study uses Conservation of resources (COR) theory to understand the relationship between work-family conflict and employee unemployment behavior (Mittal et al., 2020). The importance of the environment requires an assessment of workplace policies and work-family conflict issues and an understanding of specific social and cultural patterns of behavior (Burke et al., 2011; Foster and Ren, 2015).

**FIGURE 1:**
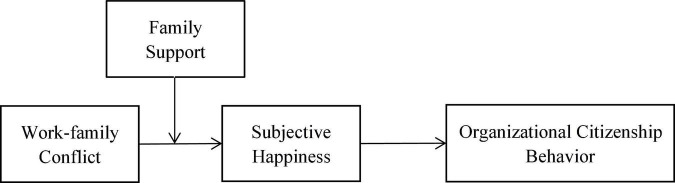
Hypothesized model of processes linking work-family conflict and organizational citizenship behavior.

To fully understand the impact of work-family conflict in China, we provide an up-to-date research dimension based on previous literature reviews. This study focuses on family support as an essential resource for female employees in mitigating work-family conflict in both work and non-work areas. This study builds a moderated mediation model that extends the theoretical knowledge of work-family conflict to the relationship of organizational citizenship behavior in the Chinese context. We investigated the role of subjective well-being in relationships and whether families support moderate relationships. In addition, the results of this study also have business application value, providing a reference for promoting the organizational citizenship behavior of female employees and addressing the challenges faced by employers and employees. Figure 1 depicts the study model.

## Hypothesis Development

### Work-Family Conflict and Organizational Citizenship Behaviors

There are different roles to play in an adult’s life, yet work and family are important in people’s lives. Because adults frequently switch positions, conflict between work and family is inevitable. For example, to complete work tasks, employees will inevitably put their energy into the work field, which will lead to insufficient time for family life, resulting in conflicts between work and family. In addition, adults will also bring the negative emotions generated by work-family conflict to work, thus affecting employees’ work behavior. Therefore, reducing such conflicts and their adverse effects has become a common concern of society (Chun and Aobo, 2020). Kahnetal and Greenhaust believe that the conflict between work and family is a conflict between roles, which will increase the pressure on work and family (Maertz et al., 2019). Moreover, work-family conflict can significantly negatively impact employees’ work performance. From this perspective, work-family conflict has a crucial effect on employees’ work behavior. As a result, this paper speculates that work-family conflict is negatively correlated with employees’ out-of-work behavior.

Organizational citizenship behaviors (OCB) is not included in the organization’s remuneration system, and employees’ behavior enhances the organization’s social image without obtaining rewards (Maertz et al., 2019). With the continuous development of the social economy and intensified market competition, organizations require employees to perform formal job responsibilities (Geng et al., 2021). They need employees to actively engage in behaviors outside their jobs (Vossen and Hofmans, 2021). Organizational citizenship behavior requires employees to invest their emotion, effort, and other resources (Taylor et al., 2009). When those resources are scarce, employees develop internal tensions, which will reduce employees’ enthusiasm and helping behavior toward leaders and colleagues (Zhang et al., 2022), thereby affecting work. According to the COR proposed by Hobfoll (Maertz et al., 2019), it can be concluded that the conflict between employees’ work and family will continue to drain employees’ resources, such as energy and time, resulting in a decrease in job satisfaction and employee happiness (Wang et al., 2022). Therefore, work-family conflict may reduce employees’ organizational citizenship behavior, which is negatively correlated with organizational citizenship behavior.

*H1:* Female professional employees’ work-family conflict is negatively related to their organizational citizenship behavior.

### The Role of Happiness

With the acceleration of the times, employees face higher requirements and expectations from the organization and enormous stress from family and personal life (Zhang et al., 2021). Therefore, some early scholars studied the related psychological factors that affect employees’ lives and work and directly linked the influencing factors of conflict with the results of negative emotions, quality of life, subjective well-being, and other psychological attitudes (Edwards and Rothbard, 2000). However, this paper mainly studies the relationship between work-family conflict and psychological factors of happiness. This paper discusses the influence of work-family conflict on employees from promoting and maintaining employees’ happiness.

Happiness is a comprehensive psychological indicator that measures the quality of individual and social life (He et al., 2000). It reflects people’s adaptability to society and reflects individuals’ sense of life satisfaction and happiness (Quan et al., 2021). In other words, happiness is the life goal that everyone aspires to and pursues all their lives, but human energy and time are limited, especially for women. Therefore, in today’s society, the conflict between work and family has become a widespread social phenomenon that impacts both employees and organizations. However, the critical reason for this phenomenon is precisely because of the resource consumption of individual employees. COR was proposed by Hobfoll (Maertz et al., 2019); it can be concluded that the conflict between employees’ work and family will continue to drain employees’ resources, such as energy and time, resulting in the decline of job satisfaction and employee happiness. From the perspective of resources, family happiness is vital emotional sustenance for employees and necessary to improve their enthusiasm for work and cope with various difficulties (Yinbin et al., 2017). Thus, happiness can be viewed as an emotional resource. When work and family are not coordinated, subjective well-being will show an apparent negative growth trend, and personal emotional resources will be severely depleted (Hoffman et al., 2007). Therefore, people will reduce the reuse of resources to avoid excessive consumption of resources. However, because people are constantly switching between work and family roles, it leads people to bring their emotions from home to work or from work to home. When resource consumption is more serious, employees will reduce their commitment to organizational behavior because of the loss of happiness (Hoobler et al., 2009). Because employees have no resources to waste on non-essential roles or things, they will pay more attention to things that cause their happiness to plummet. Moreover, according to self-determination theory and the theory of planned behavior, it is found that when the organization gives employees more opportunities for decision-making and autonomous choice, it is easy to motivate employees to work and improve organizational performance (Jesse et al., 2011). At the same time, it enhances the employee’s work happiness. For example, employees can choose flexible working hours, organize remote work, and promote benefits such as salary and travel. The above incentive measures can effectively enhance employees’ recognition of the organization and can also further improve employees’ work enthusiasm and work efficiently to ensure corresponding rewards at the work level. The reward-based incentive method can further relieve the pressure on female employees due to changing roles and improve the happiness of female employees. According to the above, good subjective well-being is produced in a harmonious atmosphere between work and family. Moreover, the personal happiness derived from employees will prompt employees to take the initiative to undertake some work or tasks outside the organization.

*H2:* Female professional employees’ subjective happiness mediates the relationship between work-family conflict and organizational citizenship behavior.

### The Moderating Role of Family Support

According to the research of role theory and boundary theory, the leading cause of work-family conflict is that when individuals switch roles in work and family, the two roles cannot adapt to each other (Khamisa et al., 2015). This incompatibility problem between roles can lead to blurred roles for female employees in these two fields, resulting in a role load that takes time and energy. In other words, people shift from family roles to work roles when they work. People need to switch from family roles such as partners, parents, and children to work roles such as employees, supervisors, and leaders; when employees leave work, they need to switch roles again. The way and outcomes people deal with reconciling work and home boundaries can affect the balance between work and home. On the other hand, if women have a high identification with a role in a specific field when the role of another field penetrates this field, they will have a strong sense of occupation and conflict. This sense of occupation also leads to work and family conflict. According to relevant data, female employees generally spend more time on housework than male employees (Bai et al., 2021). Most male employees devote their energies and resources to the work field and are less involved in family affairs (Klotz et al., 2018). Work-family conflict has little effect on male employees (Liu et al., 2013). Moreover, women will doubt their role and cause anxiety because they spend too much time in domestic labor, harming work and life, resulting in declining female employees’ happiness (Kwan et al., 2012). Therefore, to improve the well-being of female workers and coordinate the conflict between work and family, we need higher family support. When there is tremendous pressure on work and family, women need family support the most. Being able to obtain corresponding support from all aspects of the family, especially when female employees can accurately sense and understand the support provided by the family, such as the care of their husband and the understanding of their children, will buffer the fatigue and frustration brought by pressure to deal with the negative emotions brought by stress effectively (Heller et al., 2004). In addition, women are more emotional than men, which will also be why female workers are more affected by family support factors. The family support of female employees will make women feel trusted, which will make women have positive emotions and be more willing to pay for their family and work (Judge et al., 2006).

*H3:* Family support moderates the relationship between work-family conflict and happiness, and the higher the family support is, the weaker the relationship between work-family conflict and happiness.

### Family Support Increased OCB

Family support has a noticeable effect on resolving the negative emotions of female employees. When stressful events occur at work and home, they can effectively reduce the adverse effects of stress and improve female employees’ happiness (Cates et al., 2010). On the one hand, the support of the family also represents family members’ sense of identity with each other (Jeffrey and Nancy, 2000). The essence of this relationship also proves that the individual devotes his personal goals, attitudes, and behaviors to the other party in the connection and cares about the other party’s interests (Hirschi et al., 2019). Female employees with family support will pay more attention to their roles, constantly focusing on improving themselves and paying more attention to the interests of others at work and home (Kruglanski and Orehek, 2007). Therefore, when female employees consume their emotional resources, they will effectively interact and cooperate with all family members through family support to obtain more valuable resources to deal with work-family conflicts. For example, because of the support and help of her husband, the mood of female employees who have initially been in a negative attitude will be relieved. Then, the supported woman will be delighted, and her sense of happiness will be improved, so she will make behaviors conducive to her family and work (Poposki, 2011). Therefore, female employees who receive family support tend to act altruistically because of their increasing subjective well-being. Although this type of behavior reduces employee’s time and other resources, it increases their emotional resources (Radcliffe and Cassell, 2014). This kind of relationship identification will help female employees cope with the conflict between family and work and improve employees’ organizational citizenship behavior.

*H4:* Female professional employees’ family support moderates the mediating effect of work-family conflict on OCB through subjective happiness such that the indirect relationship is weaker when family support is higher.

## Materials and Methods

### Sample

We collected data using a questionnaire through a training institution in East China. We sent out questionnaires to participants who are taking business training courses that have been fully researched in past academic presentations (e.g., Peng and Xie, 2016). All participants received a monetary reward after completing the questionnaire. Before sending the questionnaire, we ensured that participants would participate anonymously, and the final survey results would only be used in academic research. This sampling strategy has proven to be a reliable way to collect data (Meyerson and Tryon, 2003; Madrid and Patterson, 2016). Small meaningful gifts were distributed to participants when the questionnaire was returned. A total of 400 company employees participated in the study, and 386 valid questionnaires were obtained, excluding invalid questionnaires such as those in which most questions were not answered. The effective recovery rate is 96.5%, which suits requirements of our research. The participants were mostly employees from 9 companies in Shanghai, Nanjing and Suzhou, excluding 204 male employees. The average age and monthly income of the samples were 31.39 and 11,736.85, respectively.

### Measures

In this study, all scales were initially studied using English. According to the procedure of Brislin (1970), we translated all the projects into Chinese for the convenience of subsequent research and investigation. The scale is derived from the results of the questionnaire. After the index system of the scale was perfected, the questionnaire was completed. The questionnaire consists of three parts. First, we need to investigate the basic situation of the samples. Second, scale needs to be revised according to 5-point Likert scale ranging from 1 (strongly disagree) to 5 (strongly agree).

#### Family Support

In terms of family support, we used the one-item scale, that is, “I get the emotional help and support I need from my family.”

#### Work-Family Conflict

Work-family conflict was assessed with the four-item scale developed by Grzywacz and Marks (2000). A sample item is, “Your job makes you feel too tired to do the things that need attention at home.”

#### OCB

To measure participants’ organizational citizenship behavior, we used a six-item scale adapted from Den Hartog et al. (2007) that included items such as “I volunteer to do things for the organization.” The Cronbach’s alpha coefficient was 0.78.

#### Individual Happiness

For the individual happiness measurement, the scale consists of four-item revised by Lyubomirsky and Lepper (1999), for example, “In general, I consider myself: 1 not a very happy person; 7 a very happy person.”

#### Control Variables

Several control variables were introduced to our analyses to minimize the potential effects of exogenous variables. More specifically, the participants’ age, marital status and salary were controlled for in this study.

## Results

### Preliminary Analyses

The mean value, standard deviation and Pearson correlation of the study variables are shown in Table 1. In terms of the descriptive analysis, age and monthly income are the primary factors with standard deviation of 4.73 and 24,790.50, respectively. This was followed by work-family conflict with a standard deviation of 0.87. Moreover, the standard deviation of family support is 0.73, which is the least popular factor affecting OCB. In the field of correlation matrix. Gender is the only variable that is not correlated with OCB. Work-family conflict was also significant but not positively correlated with subjective happiness (*r* = -0.39, *p* < 0.01). However, in terms of subjective happiness, the connection between subjective happiness and OCB (*r* = 0.42, *p* < 0.01) is positive and significant. Family support, as a form of mediating variable, is positive but not significantly correlated with subjective happiness (*r* = 0.12). In addition, age is not significantly but positively connected with OCB (*r* = 0.01). Interestingly, marital status is positive and changes with age (*r* = 0.56, *p* < 0.01). Monthly income is not significant but positively correlated with marital status (*r* = 0.13). Overall, there is no double collinearity between variables, so regression analysis can be conducted. Together, these results confirm and support our hypothesis.

**TABLE 1:** Descriptive statistics and correlations between the variables.

Variable	Mean	*SD*	1	2	3	4	5	6	7
1. Work-family conflict	2.91	0.87	–						
2. Subjective happiness	3.74	0.69	–0.39**	–					
3. Family support	4.42	0.73	–0.12	0.12	–				
4. OCB	3.98	0.61	–0.22**	0.42**	0.07	–			
5. Age	31.39	4.73	–0.07	–0.09	0.06	0.01	–		
6. Marital status	2.56	0.77	–0.13	0.14*	0.06	0.05	0.56**	–	
7. Monthly income	11,736.85	24,790.50	0.04	0.00	–0.03	–0.08	0.08	0.13	–

N = 193. *p < 0.05. **p < 0.01.

### Hypotheses Testing

To test the accuracy of our hypothesis, we adopted multiple regression analyses (Baron and Kenny, 1986; Hox, 2010). The results are shown in Table 2. Hypothesis 1 proposed that female professional employees’ work-family conflict is negatively related to their organizational citizenship behavior. After controlling age, marital status and monthly income, in model 2, work-family conflict is not positively but significantly correlated with OCB (β = –0.22; *p* < 0.01) and in model 3, work-family conflict is negatively and not significantly correlated with OCB (β = –0.06). Thus, hypothesis 1 is supported.

**TABLE 2:** Results of the moderated regression analyses.

Variable	OCB		
	Model1	Model2	Model3
Control variables			
Age	–0.03	0.03	0.01
Marital status	0.08	0.05	–0.04
Monthly income	–0.09	–0.07	–0.08
Independent variable			
Work-family conflict		–0.22**	–0.06
Mediator			
Subjective happiness			0.41***
*R* ^2^	0.01	0.06	0.19
*F*	0.68	2.81	8.66

N = 193. **p < 0.01. ***p < 0.001.

Hypothesis 2 shows that female employees’ happiness plays a mediating role in the relationship between work-family conflict and employees’ out-of-work behavior. In Model 3 of Table 2 indicates that after we added subjective happiness, as a form of mediator, subjective happiness had a good influence on OCB (β = 0.41; *p* < 0.001). In view of these results, hypothesis 2 can be supported.

**FIGURE 2:**
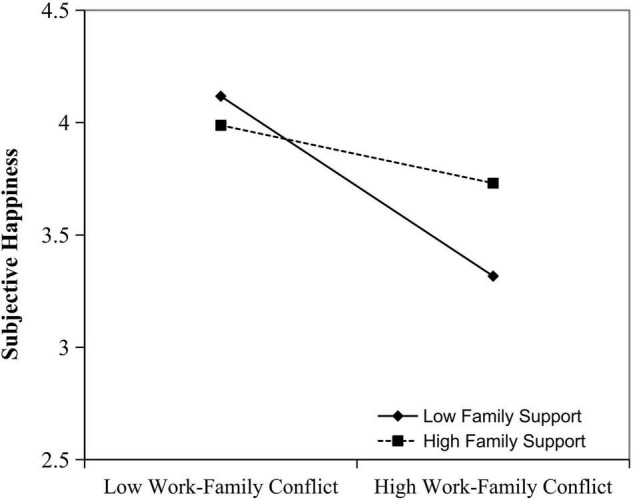
Family support as a moderator in the relationship between work-family conflict and subjective happiness.

Furthermore, hypothesis 3 showed that female professional employees’ family support moderates the relationship between work-family conflict and happiness. High family support can alleviate the negative effect of work-family conflict, promoting subjective happiness. In Figure 2, family support negatively moderates the effect of work-family conflict on subjective happiness. Having high family support, subjective happiness declines less based on work-family conflict. In light of these results, hypothesis 3 is supported.

Last, hypothesis 4 predicted that female professional employees’ family support moderates the mediating effect of work-family conflict on OCB through subjective happiness such that the indirect relationship is weaker when family support is higher. We conducted process analysis to further validate the mediating effect. Specifically, we conducted a bootstrap analysis using the PROCESS macro (Hayes, 2013) to validate the mediating role of subjective happiness. The 95% confidence interval did not contain zero (LLCI = –0.18, ULCI = –0.05), and the coefficient was –0.11, indicating that the mediating effect is significant. Thus, hypothesis 4 is supported.

## Discussion

The main purpose of this paper is to explain the relationship between work-family dissonance and organizational citizenship behavior. This study makes a thorough inquiry into the relationship by conducting a moderated mediation model. This study shows that female employees with work-family conflict are less willing to organize citizenship behavior, and their subjective well-being mediates this relationship. Additionally, we verify that the impact of work-family dissonance on organizational citizenship behavior through subjective happiness is moderated by female employees’ family support. For female employees with more family support, the less their work-family conflict is, the higher the level of happiness they perceive. Consequently, they are more likely to engage in additional employee behaviors that help the organization. In contrast, for female employees with less family support, work-family conflict can restrain their happiness and organizational citizenship behavior engagement.

### Theoretical Implications

This paper has specific theoretical significance for the following aspects. First, this study constructed an integrated moderated meditation model to further understand the impact of work-family conflict on female employees’ well-being and thus organizational citizenship behavior. This theoretical study fully proves that family support effectively alleviates the negative effect of work-family conflict on happiness, which in turn enhances the willingness to exhibit high levels of organizational citizenship behavior. Second, COR theory is used to prove the role of happiness as an intermediary. Based on the fact that individual differences is regarded as a resource, this paper hopes that can affect individuals’ response to resource consumption.

### Practical Implications

The conclusion of this study has profound practical significance for Chinese employees and managers. The increasing pressure of social achievements and family responsibilities doubled the burden of working women and their role conflict with the rapid development of China (Wang and Peng, 2017). Since the personal conflicts and responsibilities of employees in the family will affect the completion of work tasks (Kim and Hwang, 2012), role conflicts will reduce their efficiency in the family and workplace (Gou et al., 2013). Female employees can often communicate with family members and obtain adequate support to reduce the negative impact of work and family life pressure on their subjective well-being and participate in civic behavior. Considering the associations of work and family, managers in organizations should try to understand the mechanisms bred from work-family culture that can enhance a balance between female employees’ work and non-work activities. Managers should be aware of the importance of family support and provide more autonomy and feedback for female employees to enhance their work and life quality. For instance, supervisors provide exchange resources such as guidance, respect and understanding to subordinates in high-quality leader–member exchange. Subordinates may reward the leader by increasing the organization’s other social behaviors (Zhao et al., 2019).

## Limitations and Future Research Directions

Obviously, this paper also has some deficiencies, which need to be improved upon through future studies. First, this study uses a cross-sectional design, which limites the ability to establish causal direction from the resulting data. Researchers might consider conducting longitudinal designs or more complex research designs to facilitate more casual evaluations for future studies. Second, the traditional methods used in this study are mainly derived from Western cultural traditions, while the connotation of work-family conflict with distinct Chinese cultural backgrounds is different in the West (Wang and Peng, 2017; Lin et al., 2021). The development and application of more fitting scales based on the Chinese context is needed. Third, this study has identified the associated variables, while previous studies suggest variables such as organizational justice, personality, stress, overload, or fatigue to understand employees’ willingness to engage in organizational citizenship behavior (Organ et al., 2006). Future studies should examine whether there are further mediating variables that can explain the relationship between work-family conflict and organizational citizenship behavior.

## Data Availability Statement

The raw data supporting the conclusions of this article will be made available by the authors, without undue reservation.

## Ethics Statement

Ethical review and approval was not required for the study on human participants in accordance with the local legislation and institutional requirements. The patients/participants provided their written informed consent to participate in this study.

## Author Contributions

YP developed the theoretical framework and worked on literature review and manuscript writing. NA developed the theoretical framework and manuscript writing. QL worked on data collection and analysis and manuscript writing. YJ and SR worked on the revision and research design. All authors contributed to the article and approved the submitted version.

## Conflict of Interest

SR is an employee of ITO Bussiness Group, Sunyard Technology Co., Ltd. The remaining authors declare that the research was conducted in the absence of any commercial or financial relationships that could be construed as a potential conflict of interest.

## Publisher’s Note

All claims expressed in this article are solely those of the authors and do not necessarily represent those of their affiliated organizations, or those of the publisher, the editors and the reviewers. Any product that may be evaluated in this article, or claim that may be made by its manufacturer, is not guaranteed or endorsed by the publisher.
